# CaMKII/calpain interaction mediates ischemia/reperfusion injury in isolated rat hearts

**DOI:** 10.1038/s41419-020-2605-y

**Published:** 2020-05-21

**Authors:** Hong-Ting Lu, Ren-Qian Feng, Jia-Kun Tang, Jing-Jun Zhou, Feng Gao, Jun Ren

**Affiliations:** 1 Department of Physiology and Pathophysiology, The Fourth Military Medical University, Xi’an, 710032 China; 2 Department of Aerospace Medicine, The Fourth Military Medical University, Xi’an, 710032 China; 3 Center for Cardiovascular Research and Alternative Medicine, University of Wyoming, Laramie, WY 82071 USA

**Keywords:** Pharmacology, Experimental models of disease

## Abstract

Previous studies indicated that Ca^2+^/calmodulin-dependent kinase II (CaMKII), a kinase involved in the modulation of ryanodine receptor activity, activates Ca^2+^-regulated protease μ-calpain to promote myocardial ischemia/reperfusion injury. This study was performed to explore the underlying mechanisms in CaMKII-induced calpain activation to better understand heart injury. To examine the Ca^2+^ paradox and ischemia/reperfusion injury, isolated rat hearts were subjected to a Ca^2+^-free solution for 3 min, or left coronary artery occlusion for 40 min, prior to restoration of normal perfusion. Blockade of trans-sarcoplasmic reticulum Ca^2+^ flux using ryanodine and thapsigargin failed to prevent Ca^2+^ paradox-induced heart injury. In contrast, the Ca^2+^ paradox increased CaMKII auto-phosphorylation at Thr^287^, while the CaMKII inhibitor KN-62 and the Na^+^/Ca^2+^ exchanger inhibitor KB-R7943 alleviated heart injury and calpain activity. Intriguingly, the binding of μ-calpain large subunit calpain-1 (CAPN1) to phospho-CaMKII was blunted by both inhibitors. Thus, a Ca^2+^ leak via the ryanodine receptor is not an essential element in CaMKII-elicited calpain activation. In hearts receiving vector injection, ischemia/reperfusion caused elevated calpain activity and α-fodrin degradation, along with membrane integrity damage, similar to the effects noted in control hearts. Importantly, all these alterations were diminished with delivery of adeno-associated virus expressing mutant CaMKIIδC T287A. Ischemia/reperfusion increased CaMKII auto-phosphorylation and binding of CAPN1 to phospho-CaMKII, and facilitated the translocation of phospho-CaMKII and CAPN1 to the plasma membrane, all of which were reversed by injecting CaMKII mutant. Furthermore, the relocation capacity and the interaction of CaMKII with CAPN1 appeared to be dependent upon CaMKII autophosphorylation, as its mutant delivery increased the level of CaMKII, but did not increase membrane content of CaMKII and CAPN1, or their interactions. Together, CaMKII/calpain interaction represents a new avenue for mediating myocardial ischemia/reperfusion injury, and CaMKII likely serves as both a kinase and a carrier, thereby promoting calpain membrane translocation and activation.

## Introduction

Myocardial ischemia/reperfusion injury remains an unresolved medical problem and imposes a heavy healthcare burden^1^. Ischemia/reperfusion injury induces cell death, arrhythmia, and cardiac dysfunction^1^. Ca^2+^ is essential to cardiac homeostasis. Ca^2+^ handling is orchestrated by an array of regulatory membrane and sarcolemmal proteins, including voltage-gated Ca^2+^ channel, reverse-mode Na^+^/Ca^2+^ exchange, Ca^2+^-ATPase on plasma membrane, the ryanodine receptor, Ca^2+^-ATPase on sarcoplasmic reticulum (SR), and Ca^2+^ uniportor on mitochondria^2^. Tightly regulated intracellular Ca^2+^ levels govern cardiac contraction in physiological conditions, whereas intracellular Ca^2+^ overload triggers cell death in pathophysiological states^3^. Multiple lines of evidence have indicated that facilitated reverse-mode Na^+^/Ca^2+^ exchange participates in myocardial ischemia/reperfusion injury^4,5^.

The ryanodine receptor is a Ca^2+^-gated channel located on SR. Upon external Ca^2+^ stimulation, ryanodine receptors release Ca^2+^ from the SR to provoke systolic cytosolic Ca^2+^ peaks and maintain cardiomyocyte contractility^6^. Nonetheless, aberrant intracellular Ca^2+^ may leak through the ryanodine receptor to provoke cell death in pathological conditions such as myocardial ischemia/reperfusion injury^6–8^. Ryanodine receptor activity is tightly regulated by Ca^2+^/calmodulin-dependent protein kinase II (CaMKII), a serine/threonine kinase containing catalytic, autoinhibitory, and Ca^2+^/calmodulin binding domains. CaMKIIδ is the predominant cardiac isoform^9^. Once Ca^2+^/calmodulin bound, CaMKII undergoes autophosphorylation at the Thr^287^ residue in the autoinhibitory domain and remains persistently activated even after dissociation from Ca^2+^/calmodulin. CaMKII is reported to be active during the early phase of postischemic reperfusion^5,10^. Previous studies have shown that CaMKII phosphorylates the ryanodine receptor at the S2814 residue in the SR, and promotes Ca^2+^ leak from the SR, ultimately contributing to cell death and cardiac dysfunction^8,11,12^. To this end, it is well known that the Na^+^/Ca^2+^ exchanger-CaMKII-ryanodine receptor cascade plays a central role in Ca^2+^ overload-induced injury in ischemic/reperfused hearts.

Ample evidence also suggests that calpain plays a vital role in ischemia/reperfusion injury. Calpain is a Ca^2+^-dependent protease, and the calpain superfamily possesses 15 genes in the human genome^13^. Several studies have revealed that over-activated calpain increases persistent Na^+^ current via cleaving the Na^+^ channel, stimulates protein kinase C, and destroys skeleton membrane proteins, α-fodrin and ankyrin-B, resulting in abnormal cell function^10,14–17^. μ-calpain, a heterodimer consisting of a large catalytic subunit calpain-1 (CAPN1) and a small regulatory subunit, is present in the heart. We and others have previously demonstrated that upon stress, μ-calpain becomes activated after its translocation to the sarcolemmal membrane. More importantly, inhibition of μ-calpain protects against heart injury^18,19^. Given the pivotal role of Ca^2+^ leak from SR in this scenario, μ-calpain activation is suspected to be a secondary effect.

Interestingly, evidence supports a more direct activation modality of μ-calpain, namely: (1) calpain destroys the ryanodine receptor during ischemia/reperfusion^20–22^, and (2) calpain activity is regulated by phosphorylation modification, including protein kinase A, protein kinase C, and mitogen-activated protein kinase^23–25^. Furthermore, recent studies have demonstrated that CaMKII possesses other biological functions, such as serving as a carrier or structural component for substrate transmission^26,27^. These observations prompt us to speculate that the Na^+^/Ca^2+^ exchanger–CaMKII–calpain axis may be a primary route mediating Ca^2+^ overload-induced tissue injury in ischemic/reperfused hearts.

To this end, this study was designed to explore the underlying mechanisms in CaMKII-induced calpain activation in order to help elucidate the possible mechanism behind myocardial ischemia/reperfusion injury. To accomplish this, we conducted Ca^2+^ paradox experiments. The Ca^2+^ paradox occurs when hearts are perfused with a Ca^2+^-containing solution after a period of perfusion with a Ca^2+^-free solution, ultimately leading to Ca^2+^ influx through reverse-mode Na^+^/Ca^2+^ exchange, activation of calpain, and damage to the cellular structure^14,18^. Interestingly, Ca^2+^ transporter proteins, ryanodine receptor and sarco/endoplasmic reticulum Ca^2+^ ATPase, are not involved in this event^28^. Therefore, we evaluated CaMKII in the Ca^2+^ paradox, and tested whether CaMKII-induced calpain activation is secondary to Ca^2+^ leak through the ryanodine receptor or functions as a more direct regulatory machinery. Subsequently, co-immunoprecipitation and immunofluorescence were performed to examine the interaction between CaMKII and calpain. Finally, the CaMKII T287A mutant was employed to discern the possible mechanism of action behind CaMKII-induced calpain activation.

## Materials and methods

### Chemicals and antibodies

KN-62 (1277), KB-R7943 (1244), ryanodine (1329), and thapsigargin (1138) were purchased from Tocris Bioscience (Bristol, UK). Antibodies against cytochrome c (4272), CaMKII (4436 and ab134041), phospho-CaMKII (12716 and ab171095), CAPN1 (2556, c5736, and ab39170), Flag (F7425), cytochrome oxidase subunit IV (COX IV, ab202554), sodium/hydrogen exchanger 1 (NHE1, Chemicon AB3081), Na^+^/K^+^-ATPase α1 (23565), and GAPDH (2118) were obtained from Cell Signaling Technology (Beverly, MA, USA), Abcam (Cambridge, MA, USA) and Sigma (Saint Louis, MO, USA), respectively. α-fodrin (BML-FG6090) and tetramethylrhodamine (TRITC, T-2769), or Alexa Fluor^®^488 (A32723)-conjugated secondary antibodies, were acquired from Enzo Life Sciences (Plymouth Meeting, PA, USA) and Thermo Fisher Scientific (Eugene, OR, USA), respectively. The assay kits for LDH (KA0878), calpain (QIA120), and caspase-3 (K106-100) activity were provided by Abnova (Taipei, Taiwan), Calbiochem (San Diego, CA, USA) and Biovision (Milpitas, CA, USA), respectively. The Mem-PER^TM^ Plus Kit (89842) and Pierce^TM^ BCA protein assay kit (23227) were from Thermo Scientific (Rockford, IL, USA). All other chemicals were purchased from Sigma (Shanghai, China).

### Animals

Male Sprague Dawley rats weighing 200–250 g were provided by the Animal Center of the Fourth Military Medical University (Xi’an, China). Animals were anesthetized via intraperitoneal administration of 3% pentobarbital sodium (60 mg/kg b. w.) before isolated heart preparation and in vivo experiments, as described previously^14,29^. All animal experiments were approved by the Institutional Animal Care and Use Committee of the Fourth Military Medical University, and all animal procedures complied with the *Guide for the Care and Use of Laboratory Animals (the 8*^*th*^ *edition)* issued by the National Research Council of the United States.

### Virus injection

The cDNA of the CaMKII δC gene was synthesized based on the known cDNA sequence^9,30^. The construction of recombinant adeno-associated virus (AAV) type 9 expression vector pHBAAV-CMV-MCS-3×Flag-T2A-GFP with CaMKII δC mutant T287A was performed by Hanbio Technology Co. Ltd. (Shanghai, China). The green fluorescent protein (GFP) and Flag-tagged CaMKII δC mutant were linked by a self-cleaving T2A peptide sequence, which mediates co-expression of the reporter gene GFP with the target cDNA CaMKII independently^31^. Viral concentration was determined to be 1.2 × 10^12^ viral particles per mL. The viral infection was carried out using a technique described previously^29^. In brief, a respiratory mask was placed on the rat’s face with 60 breaths per min and 7 mL per breath after anesthesia. The left chest and intercostal muscles between the third and fourth ribs were separated via blunt dissection. Ribs were then cut off after ligation, and the heart was exposed by pulling the ligatures out. Two sites in each side of the left descending coronary received an injection with a separation of about 2–3 mm. The injection volume for each site was 15 μL. The chest was then closed and sutured. All experiments were performed three weeks later.

### Heart perfusion and experimental protocol

Isolated heart perfusions were performed in accordance with routine laboratory methods^10,18^. Briefly, after anesthetization and heparinization, the heart was quickly excised, and the aorta was connected to a Langendorff apparatus at a constant pressure of 80 mmHg at 37 °C. The Krebs–Henseleit (KH) perfusion solution contained the following (in mM): NaCl, 118; KCl, 4.7; MgSO_4_, 1.2; KH_2_PO_4_, 1.2; CaCl_2_, 1.25; NaHCO_3_, 25; and glucose, 11 (pH = 7.4, 37 °C). The perfusates were filtered and equilibrated with a gas mixture containing 95% O_2_ and 5% CO_2_. A 20 min period was given to ensure constant mechanical properties. We preestablished that any heart with a heart rate less than 250 beats/min, or coronary flow more than 15 ml/min or less than 8 ml/min at the end of equilibration would be excluded from this study. Any heart displaying arrhythmia during this period was also discarded.

In Ca^2+^ paradox studies, the hearts were, in accordance with the random number table, divided into groups as follows: (1) a control group, where the hearts received normal KH solution perfusion; (2) a Ca^2+^ paradox group, where the procedures were identical to our previous studies in which the hearts were perfused with a Ca^2+^-free solution for 3 min, followed by a normal KH solution for 30 min^14^; and (3) a Ca^2+^ paradox with drugs group, where KN-62, KB-R7943, ryanodine, and thapsigargin were introduced 1 min before and 3-min throughout the Ca^2+^-free solution perfusion, as well as 3 min after restoring normal KH solution. KN-62 at 3 μM and KB-R7943 at 10 μM were used to inhibit CaMKII and the Na^+^/Ca^2+^ exchanger, respectively^10,32–34^. Ryanodine at 10 μM and thapsigargin at 1 μM were used to block Ca^2+^ flux across the SR^28,35^. The sample size was estimated using statistical software PASS (Kaysville, UT, USA). Thirty rats (6 rats in each group) were used to determine myocardial injury area, and another 30 rats (6 rats in each group) were used for biochemical and histological studies. The results were interpreted by an examiner who was blind to group assignment.

To determine the role of CaMKII self-phosphorylation at T287 in calpain activation in myocardial ischemia/reperfusion, rat left ventricles were randomly injected with saline (control), AAV vector, and AAV expressing the CaMKIIδC T287A mutant (CaMKII-M). Three weeks later, hearts were isolated and subjected to normal KH solution perfusion as a control (sham group). Alternatively, they received 40 min regional ischemia/reperfusion (I/R group). Briefly, a 4-0 silk suture was passed around the left coronary artery close to its origin. Forty minutes of ischemia was carried out by making a slipknot to occlude the artery and reperfusion was achieved by loosening the slipknot. In this experiment, 48 rats (8 rats in each group) were used to determine myocardial injury area, and another 48 rats (8 rats in each group) were used for biochemical and histological studies.

### Measurement of myocardial injury area and enzyme activity

Following reperfusion, the left coronary artery was religated, and 1 mL 1% Evans blue was injected to delineate the nonischemic area. Heart slices were prepared and incubated with 1% triphenyltetrazolium at 37 °C for 15 min. Myocardial injury area and risk zone were evaluated quantitatively using planimetry, as described previously^10^.

Quantification of LDH was carried out following the manufacturer’s procedures. The absorbance in the reaction mixture was read at 450 nm and the content of LDH in the coronary effluent was calculated by reference to a standard curve. Caspase-3 activity was quantified by detecting the chromophore p-nitroaniline (*p*NA), which was cleaved from the substrate DEVD-*p*NA. The value was read at 405 nm. Calpain activity was measured using Suc-LLVY AMC as the substrate. The released AMC was detected by fluorimetry with 360 nm excitation and 430 nm emission filters. Activity for caspase-3 and calpain was normalized in relation to the control, as described previously^10,18^.

### Membrane integrity assessment

Five minutes after reperfusion, the hearts were perfused with 0.01% Evans blue dye for 5 min and then harvested. A 4-μm thick frozen section was prepared and washed with acetone. The tissue sections were examined with a laser-scanning confocal microscope, and the images were scanned with an FV-10-ASW system (Olympus FV1000, Tokyo, Japan). The excitation and emission wavelengths were 543 and 590 nm, respectively. A histologist who was unaware of the group assignment examined all sections, and the percentage of positive cells was calculated as described previously^10,36^.

### Immunofluorescence

As described previously^10,18^, 5 min after reperfusion, the ischemic region of the left ventricle was excised and fixed with 10% formalin. Tissue slices with 4 μM thickness were prepared. After dewaxing, the slices were stained with antibody against CAPN1 (1:100) or phospho-CaMKII at T287 (1:100) at 4 °C overnight, followed by tetramethylrhodamine isothiocyanate or Alexa Fluor^®^488-conjugated secondary antibody at 1:500. The nuclei were counterstained with 4′,6-diamidino-2-phenylindole (DAPI) at 2 μg/mL. Finally, a histologist, who was unaware of the group assignments examined all the slices with a confocal laser scanning microscope (Olympus FV1000, Tokyo, Japan). Five fields for each slide were examined under a ×600 field. The degree of co-localization of CAPN1 with CaMKII is presented as a Pearson’s correlation unit using the image analysis software Image-Pro Plus. The Pearson’s correlation unit, which varies between zero and one, represents the degree of association of pixels in different channels of the confocal images. A value of zero indicates no linear relationship, whereas one reflects a perfect linear relationship between two molecules. A value above 0.7 suggests a strong positive association. To determine fluorescent protein expression, the rats were euthanized three weeks after virus injection. Frozen sections of the hearts were prepared and examined with VS-ASW-S6 (Olympus, Tokyo, Japan).

### Immunoprecipitation and western blot

In accordance with previous studies^37,38^, left ventricular tissues were homogenized with immunoprecipitating buffer containing (in mM) Tris-HCl (pH 7.4) 20, NaCl 150, EDTA 1, sodium pyrophosphate 2.5, β-glycerophosphate 1, Na_3_VO_4_ 1, PMSF 1, and 1% Triton X-100. The lysates were centrifuged at 12,000× rpm for 30 min at 4 °C, and 800 μL of the supernatants were mixed with 10 μL of prewashed protein A sepharose beads in the presence of either 5 μL of rabbit anti-CAPN1 antibody for the experimental groups, or normal IgG for the control group. The mixtures were rotated constantly at 4 °C overnight. The beads were then collected by centrifugation at 3000× rpm for 2 min at 4 °C and washed three times to remove nonspecifically binding proteins. Next, the beads were suspended in sodium dodecyl sulfate polyacrylamide gel electrophoresis (SDS-PAGE) loading buffer and heated at 95 °C for 5 min. Finally, SDS-PAGE was performed, and CAPN1 and T287-phosphorylated CaMKII antibodies were used for immunoblot analysis. The homogenates without bead treatment were used as the input controls.

Membrane proteins were extracted in accordance with the instructions included with the Mem-PER^TM^ Plus Kit. To detect the content of cytosolic cytochrome c, the tissues were homogenized with buffer containing (in mM) Tris-HCl (pH 7.4) 50, sucrose 250, EDTA 1, DTT 1, and protease cocktail inhibitors. To measure other proteins, the tissues were homogenized with buffer containing (in mM) Tris-HCl (pH 7.4) 50, NaCl 150, EDTA 5, dithiothreitol 1, 1% Triton X-100, 1% protease inhibitor cocktail, and 1% phosphatase inhibitor cocktail. The lysates were then centrifuged at 12,000× rpm for 30 min at 4 °C. The supernatants were aliquoted, and the protein concentrations were quantified with a protein assay kit. After gel electrophoresis and protein transfer from the gel to the nitrocellulose membrane, the membranes were incubated with antibodies (1:1000) of CAPN1, CaMKII, phospho-CaMKII, cytochrome c, α-fodrin, Flag, and the α_1_ subunit of Na^+^/K^+^-ATPase at 4 °C overnight, followed by incubation with horseradish peroxidase-conjugated secondary antibody for 1 h at room temperature. Finally, the proteins were probed with chemiluminescence and quantified using Quantity One software (Bio-Rad Inc., Hertfordshire, UK), as described previously^10^. Equal protein loading was confirmed by determining levels of GAPDH, COX IV or NHE1 in each group.

### Statistical analysis

All values are expressed as the mean ± standard error of the mean. Statistical comparisons were carried out using Prism 5.0 (GraphPad Software Inc, La Jolla, CA, USA). The data exhibited normal distribution and equality of variance. A one-way ANOVA or two-way ANOVA was performed to compare the differences among several groups, as appropriate, which was followed by unpaired Student’s *t* tests. A two-tailed *P* value of less than 0.05 was considered statistically significant.

## Results

### KN-62 and KB-R7943, but not SR inhibitors ryanodine and thapsigargin, antagonized cardiac injury during the Ca^2+^ paradox and decreased the binding of CAPN1 to phospho-CaMKII

**Fig. 1:**
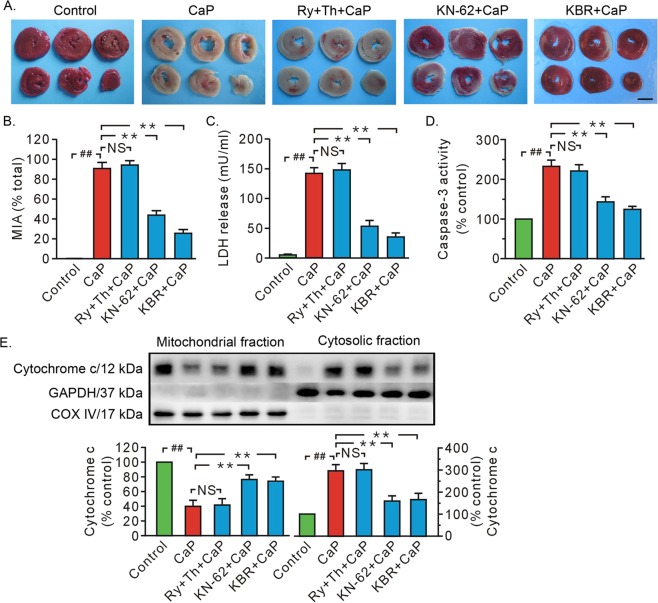
Both KN-62 and KB-R7943 ameliorated heart injury in Ca^2+^ paradox, but combined treatment with both ryanodine and thapsigargin did not. **a** Representative heart slice TTC staining. Scale bar, 0.5 cm. **b**, **c** Grouped results of myocardial injury area and LDH release in the coronary effluent, respectively. **d** Grouped results of caspase-3 activity. **e** Representative blots of cytochrome c in mitochondrial and cytosolic fraction and grouped results of densitometric analysis. GAPDH and COX IV served as a loading controls. The values are expressed as the percentages of the control values. Each bar represents the mean ± SEM; *n* = 6 rats in each group. Western blots were performed in five independent biological experiments and for three technical replicates per sample. ^##,^ **P < 0.01. NS no significant difference. CaP Ca^2+^ paradox, KBR KB-R7943, MIA myocardial injury area, Ry ryanodine, Th Thapsigargin.

The Ca^2+^ paradox caused severe cardiac injury as evidenced by remarkable rises in myocardial injury area, LDH release, caspase-3 activity, and the release of cytochrome c from the mitochondria to the cytosol (Fig. 1a–e). Given the essential role of the SR in the regulation of cytosolic Ca^2+^, we evaluated the SR’s role in Ca^2+^ paradox-induced heart injury. Our data revealed that treatment with ryanodine (10 μM) and thapsigargin (1 μM) failed to affect injured myocardial tissue size, LDH release, caspase-3 activity, or mitochondrial loss of cytochrome c (Fig. 1). In contrast, both KB-R7943 (10 μM), an inhibitor of Na^+^/Ca^2+^ exchanger, and KN-62 (3 μM), an inhibitor of CaMKII, attenuated Ca^2+^ paradox-induced cardiac injury, which manifested as reduced myocardial injury area, LDH release, and apoptosis (Fig. 1a–e).

**Fig. 2:**
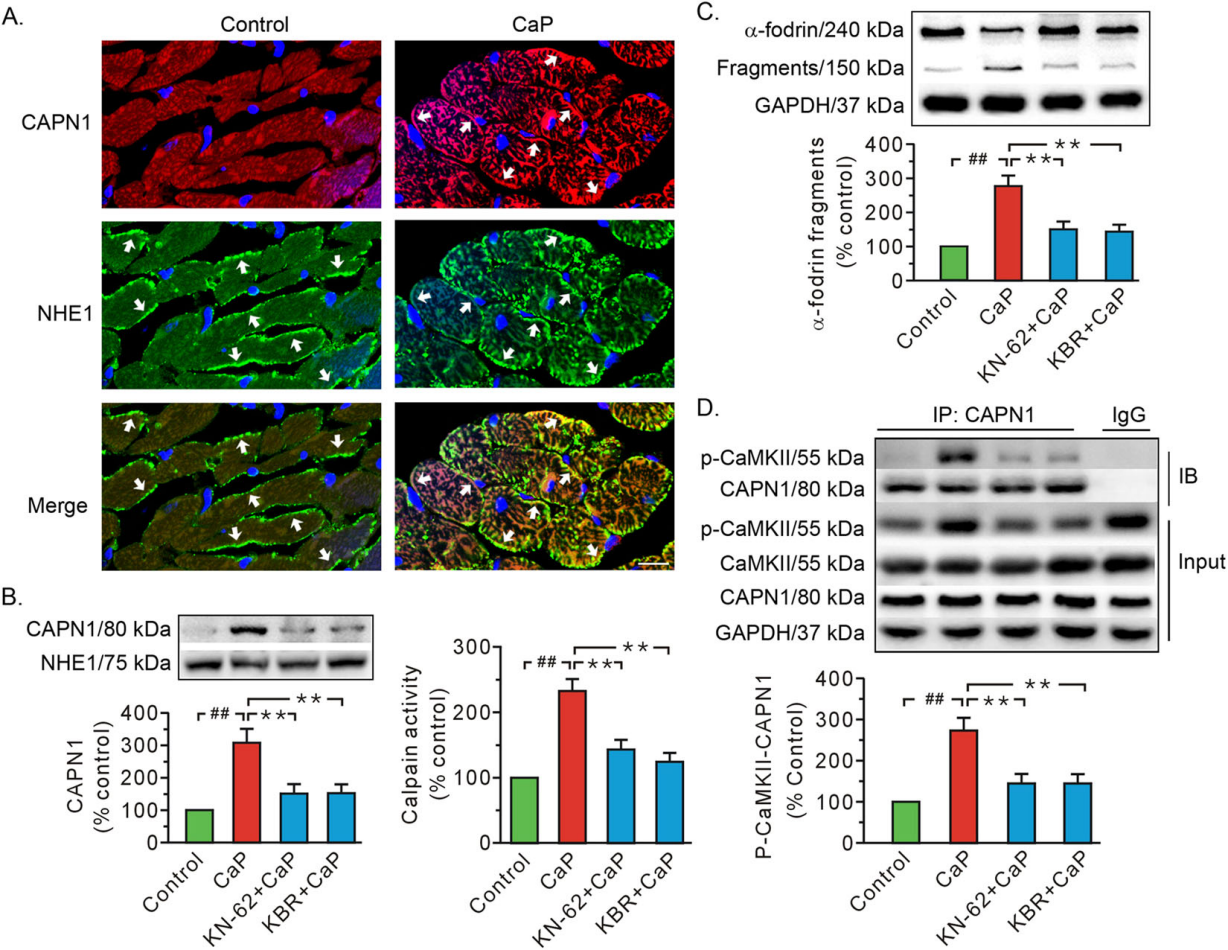
Both KN-62 and KB-R7943 impeded calpain activation and the binding of CAPN1 to T287-phosphorylated CaMKII in the Ca^2+^ paradox. **a** Representative immunofluorescence images showing CAPN1 membrane-positive cells in the Ca^2+^ paradox. Red, CAPN1; Green, NHE1, a positive control for plasma membrane protein; blue, nuclei. The arrows denote the positive signals of CAPN1 and NHE1 in the membrane. Scale bar, 10 μm. **b** Representative immunoblots for membrane CAPN1 proteins and densitometric analysis (left), along with grouped results of calpain activity (right). **c** Representative blots of full length and 150 kDa fragments of α-fodrin and grouped results of the densitometric analysis. **d** Representative immunoprecipitation and whole-cell lysate input for T287-phosphorylated CaMKII (p-CaMKII), CaMKII, and CAPN1, along with grouped analysis of T287-phosphorylated CaMKII–CAPN1 interaction. The CaP whole-cell lysates served as the input and normal IgG served as a negative control to pull-down the CAPN1 in the IgG group. NHE1 and GAPDH served as loading controls. Values (**b**, **d**) and the ratio of the fragments to total signal per lane (**c**) are expressed as the percentage of the control group. Each bar represents the mean ± SEM; *n* = 6 rats in each group. Western blots were performed in five independent biological experiments and for three technical replicates per sample. ^##,^ **P < 0.01. CaP Ca^2+^ paradox, KBR KB-R7943, IB immunoblot, IP immunoprecipitation.

The Ca^2+^ paradox increased the membrane content of CAPN1 as well as calpain activity (Fig. 2a, b). The Ca^2+^ paradox also promoted the proteolysis of calpain substrate α-fodrin (Fig. 2c). We note that all of these alterations were diminished in the presence of KN-62 and KB-R7943 (Fig. 2a–c). More importantly, the co-immunoprecipitation data indicated that the Ca^2+^ paradox increased the level of CaMKII phosphorylation in the input from whole cell lysates (Fig. 2d), as well as the binding of CAPN1 to T287-phosphorylated CaMKII, which was blocked by both KB-R7943 and KN-62 (Fig. 2d). There was no significant difference among groups in CaMKII from whole cell lysate input (Fig. 2d). These data support the conclusion that phosphorylation enhances the binding capacity of CaMKII with CAPN1.

### Mutant CaMKIIδC T287A alleviated heart injury and calpain activation

**Fig. 3:**
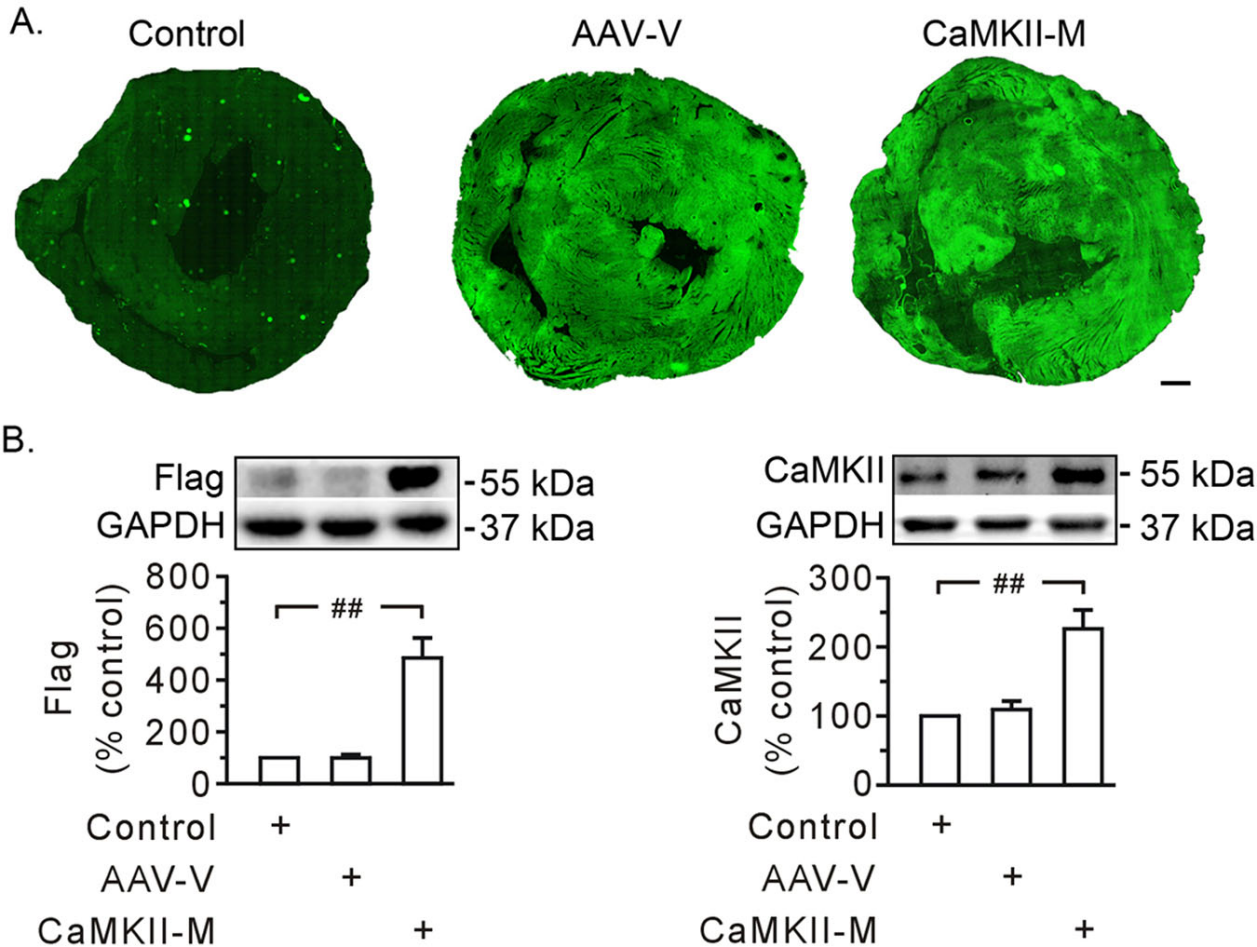
Evaluation of in vivo gene transfer. **a** Confocal images showing the expression of green fluorescent protein. Approximately, 70% of the left ventricle was stained positive for green protein (T287A mutant expression). Images from left to right are an injection of saline (control), AAV vector (AAV-V), and AAV expressing mutant CaMKIIδC T287A (CaMKII-M), respectively. Scale bar, 1 mm. **b** Representative immunoblot data showing the expression of Flag (left) and CaMKII (right) in rat hearts, along with densitometric analysis. The proteins were prepared 3 weeks after virus injection. GAPDH served as a loading control. Each bar represents the mean ± SEM; *n* = 4 rats in each group in confocal image analysis. Western blots were performed in four independent biological experiments and for three technical replicates per sample. ^##^*P* < 0.01.

**Fig. 4:**
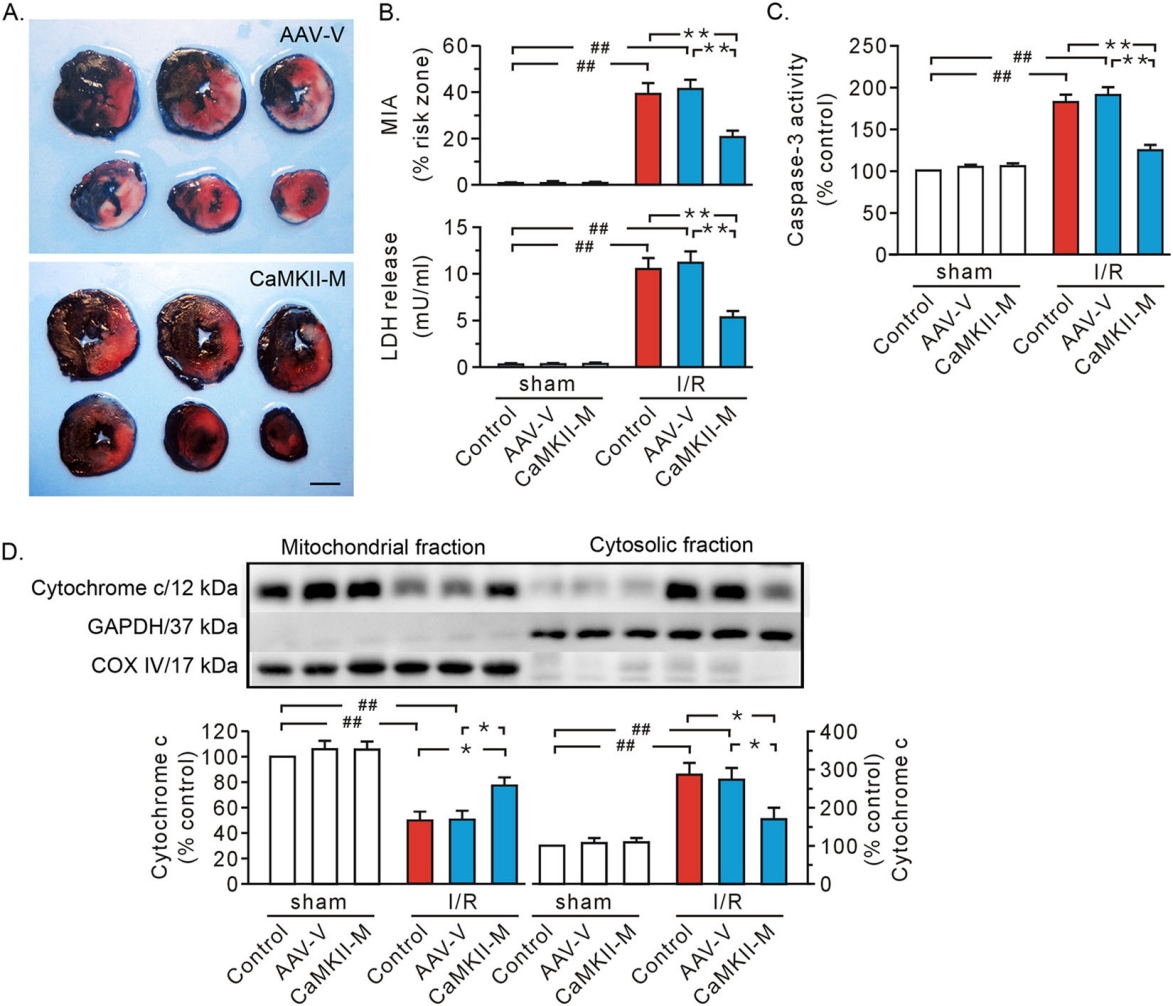
Mutant CaMKIIδC T287A protected isolated hearts from ischemia/reperfusion injury. **a** Representative heart slice TTC staining. Scale bar, 0.5 cm. **b** Grouped results of myocardial injury area (top) and LDH level in coronary effluent (bottom) respectively. **c** Grouped results of caspase-3 activity. **d** Representative blots of cytochrome c in mitochondrial and cytosolic fraction, along with grouped results of densitometric analysis. GAPDH and COX IV served as loading controls. Values are expressed as the percentage of the control in the sham group. Each bar represents the mean ± SEM, *n* = 8 rats in each group. Western blots were performed in five independent biological experiments and for three technical replicates per sample. *P < 0.05, ^##,^ **P < 0.01. I/R ischemia/reperfusion, AAV-V AAV vector, CaMKII-M AAV expressing mutant CaMKIIδC T287A, MIA myocardial injury area.

As autophosphorylation of CaMKII at T287 serves as an important step for CaMKII activity, AAV expressing CaMKIIδC T287A was utilized to discern the nature of CaMKII action. After Flag and GFP became visible 3 weeks after viral injection (Fig. 3), myocardial ischemia/reperfusion was carried out. The hearts receiving AAV vector exhibited an enlarged injured myocardial area, and an increase in LDH release, caspase-3 activity, and mitochondrial loss of cytochrome c, which were similar to the results seen in the control group (Fig. 4a–d). We found that mutant CaMKIIδC T287A significantly protected hearts against ischemia/reperfusion injury (Fig. 4a–d).

**Fig. 5:**
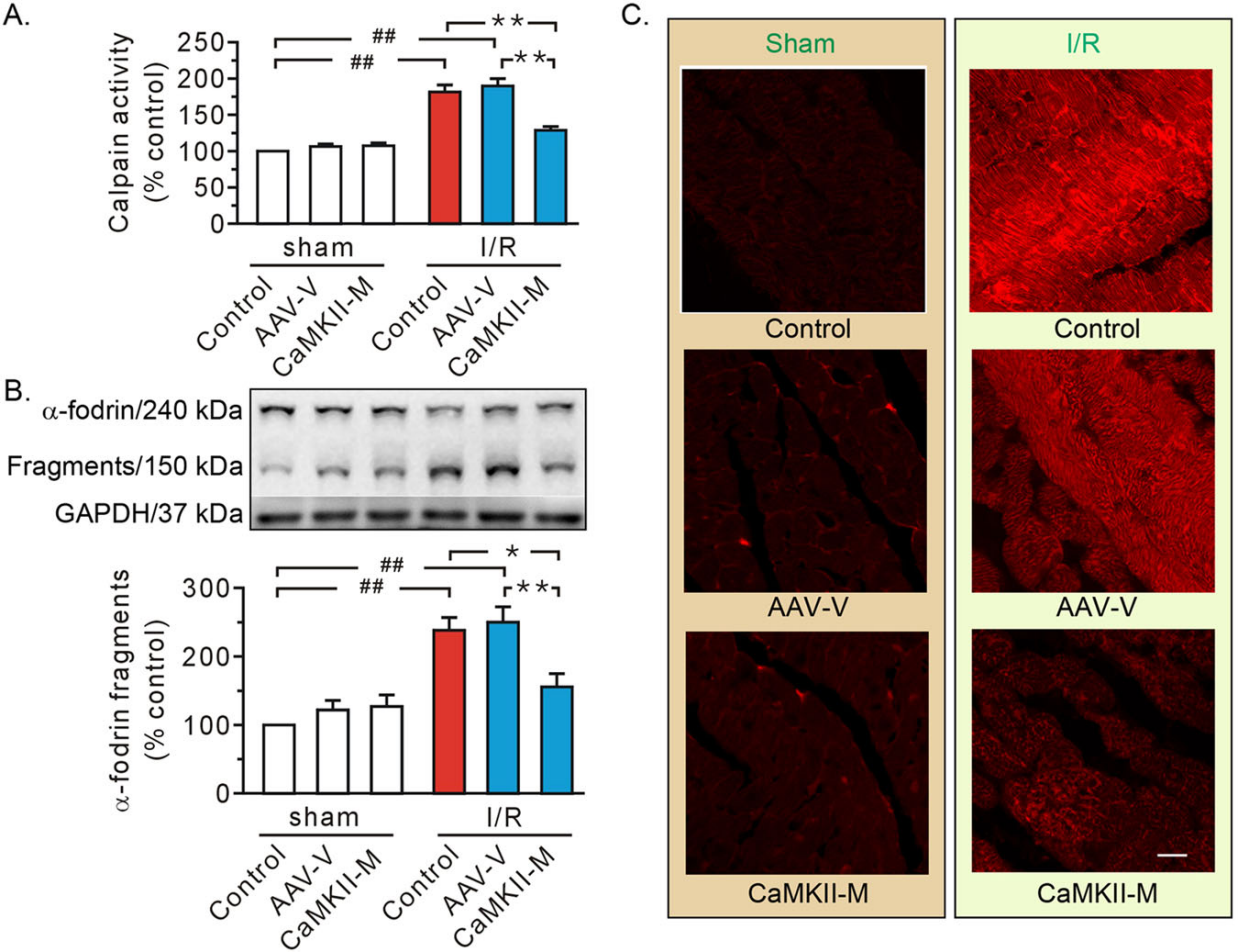
Mutant CaMKIIδC T287A inhibited calpain activity, reduced α-fodrin degradation, and improved membrane integrity. **a** Grouped results of calpain activity. Values are expressed as a percentage of the control in the sham group. **b** Representative blots of full length and 150 kDa fragments of α-fodrin, as well as grouped results of the densitometric analysis. GAPDH was used as a loading control and the ratio of the fragments to total signal per lane is expressed as a percentage of the control in the sham group. **c** Representative confocal images for Evans blue uptake. Scale bar, 25 μm. Each bar represents the mean ± SEM, *n* = 8 rats in each group. Western blots were performed in five independent biological experiments and for three technical replicates per sample. *P < 0.05, ^##,^ **P < 0.01. I/R ischemia/reperfusion, AAV-V AAV vector, CaMKII-M AAV expressing mutant CaMKIIδC T287A.

In hearts receiving AAV vector injection, ischemia/reperfusion promoted calpain activity (Fig. 5a), the proteolysis of α-fodrin (Fig. 5b), and intracellular accumulation of the Evans blue dye (Fig. 5c), a phenomenon reminiscent of control hearts. More importantly, all these alterations were diminished when injecting AAV expressing mutant CaMKIIδC T287A (Fig. 5a–c).

### CaMKIIδC T287A mutation lost its binding capacity with CAPN1

**Fig. 6:**
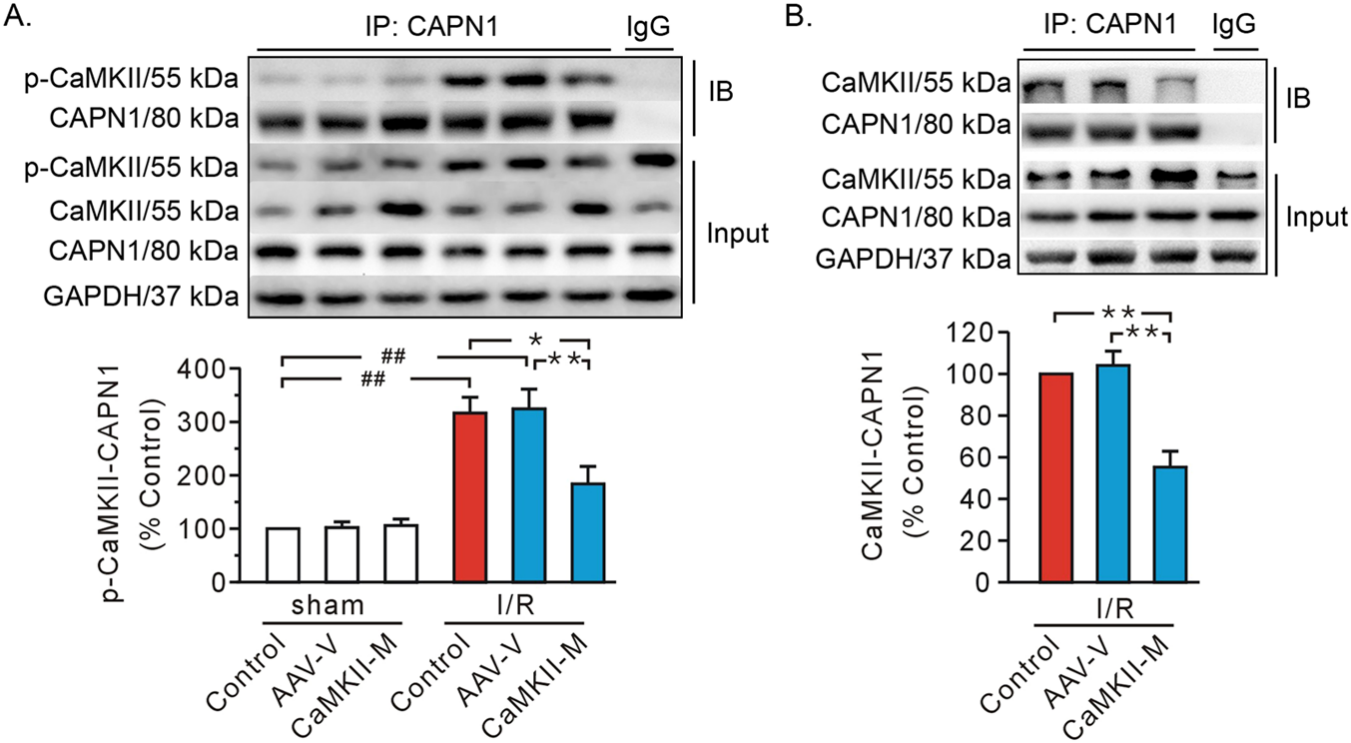
Representative immunoprecipitation and input gel blots from whole cell lysates, along with grouped analysis of their interactions. **a** The interaction between CAPN1 and T287-phosphorylated CaMKII (p-CaMKII). **b** The interaction between CAPN1 and CaMKII. The I/R whole cell lysates served as the input and normal IgG served as a negative control to pull-down the CAPN1 in the IgG group. GAPDH served as a loading control. Values are expressed as the percentage of the control group values. Each bar represents the mean ± SEM. Western blots were performed in five independent biological experiments and for three technical replicates per sample. *P < 0.05, ^##,^ **P < 0.01. AAV-V AAV vector, CaMKII-M AAV expressing mutant CaMKIIδC T287A, IB immunoblot, IP immunoprecipitation, I/R ischemia/reperfusion.

Immunoprecipitation data noted an abrupt rise of CaMKII autophosphorylation at Thr^287^ in whole-cell lysates from ischemic/reperfused hearts (Fig. 6a). The data also indicated the presence of interaction between phospho-CaMKII and CAPN1 in ischemic/reperfused hearts, both of which were attenuated with mutant CaMKIIδC T287A (Fig. 6a). These results indicate that phosphorylation of CaMKII enhances the binding of CaMKII with CAPN1. To confirm this conclusion, immunoblotting was performed using the CaMKII antibody. Delivery of mutant CaMKIIδC T287A increased the level of CaMKII in whole cell lysates (Fig. 6b). However, the interaction between CAPN1 and CaMKII (Fig. 6b) exhibited a similar trend comparable to that noted between CAPN1 and phosphorylated CaMKII (Fig. 6a). These data favor that the co-immunoprecipitation bands seen in Fig. 6b likely represent phosphorylated CaMKII. It is possible that CaMKII mutation lost the ability to interact with CAPN1, thus nullifying the binding between CAPN1 and phosphorylated CaMKII.

### CaMKIIδC T287A mutation antagonized CAPN1 membrane recruitment and dampened its translocation ability

**Fig. 7:**
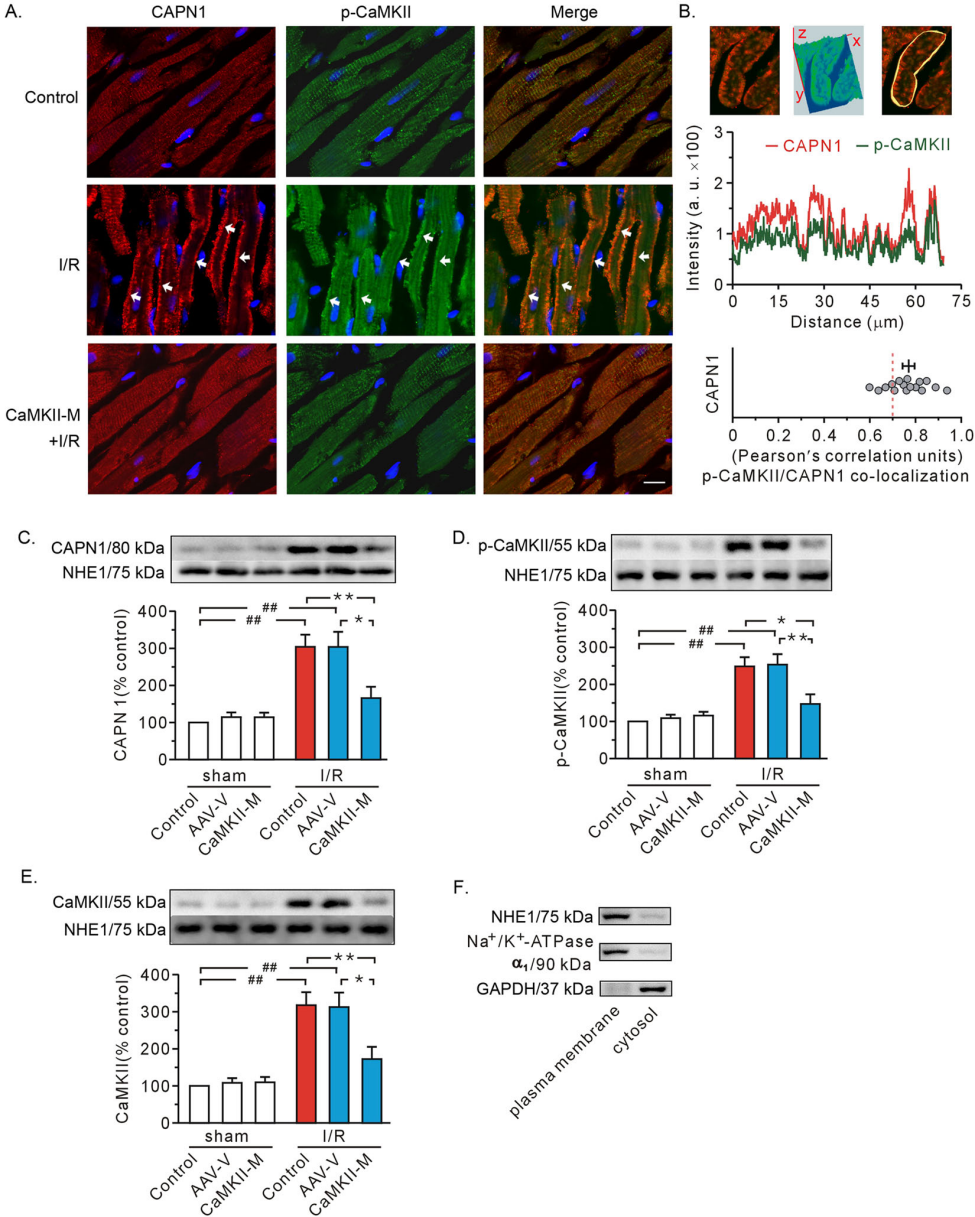
Confocal image and western blot analysis of T287-phosphorylated CaMKII (p-CaMKII) and CAPN1 on the plasma membrane. **a** Representative double-label staining. The images from the top to bottom refer to the control in the sham group, I/R, and I/R with CaMKIIδC T287A mutant respectively. Red, CAPN1; green, p-CaMKII; blue, nuclei. The arrows denote the positive signals of CAPN1 and p-CaMKII in the membrane. Scale bar, 20 μm. **b** Pearson’s correlation analysis. Top, a representative double-label positive cell (left), along with three-dimensional imaging view (middle) and the yellow region of interest (indicating co-localization, right); middle, the fluorescence intensity of both red and green channel signals corresponding to the region of interest in the top; bottom, the correlation analysis showing the co-localization degree of p-CaMKII with CAPN1. **c**–**e** Representative immunoblots for CAPN1, p-CaMKII, and CaMKII in membrane fraction, along with densitometry analysis. NHE1 served as a loading control. Values are expressed as the percentage of the control in the sham group. **f** Western blot of specific markers in the plasma membrane and cytosolic fractions. Each bar represents the mean ± SEM. For image analysis, *n* = 16 cells in 4 rat hearts. Western blots were performed in four independent biological experiments and for three technical replicates per sample. *P < 0.05, ^##,^ **P < 0.01. AAV-V AAV vector, CaMKII-M AAV expressing mutant CaMKIIδC T287A.

Double-label staining (Fig. 7a) demonstrated that ischemia/reperfusion promoted translocation of both CAPN1 and T287-phosphorylated CaMKII to the plasma membrane. Image Pro-Plus software was used to quantify co-localization of CAPN1 (red) with T287-phosphorylated CaMKII (green). Figure 7b displays a three-dimensional image exhibiting a pronounced degree of overlap between the two molecules. Once a region of interest covering the entire cell membrane was chosen, the fluorescence intensity values of both red and green channels (Fig. 7b, middle), as well as the Pearson’s correlation unit (Fig. 7b, bottom), was visible. CAPN1 exhibited a substantial degree of co-localization with T287-phosphorylated CaMKII (*r* = 0.77 ± 0.03, Fig. 7b), further corroborating the interaction between the two molecules.

Another important finding from our confocal study was that mutant CaMKIIδC T287A (CaMKII-M) overtly decreased the membrane recruitment of both CAPN1 and T287-phosphorylated CaMKII (Fig. 7a). This finding was further consolidated using western blot analysis of the plasma membrane (Fig. 7c, d). Finally, we found that injection with AAV vector exhibited few effects (Fig. 7c, d). These results suggest CaMKII may “transport” CAPN1 to the plasma membrane.

To discern if CaMKII translocation is dependent upon its phosphorylation, plasma membrane proteins were examined using a CaMKII antibody, which recognizes both non-phosphorylated and phosphorylated CaMKII. Injection of mutant CaMKIIδC T287A failed to alter membrane content of CaMKII (Fig. 7e). Moreover, the change of CaMKII level in the plasma membrane (Fig. 7e) was similar to that of phosphorylated CaMKII (Fig. 7d). These data indicate that the gel bands observed in Fig. 7e may represent phosphorylated CaMKII. We suggest that the CaMKII mutation would dampen its relocation or mobile capacity, that is, membrane translocation of CaMKII is dependent upon its phosphorylation.

## Discussion

**Fig. 8:**
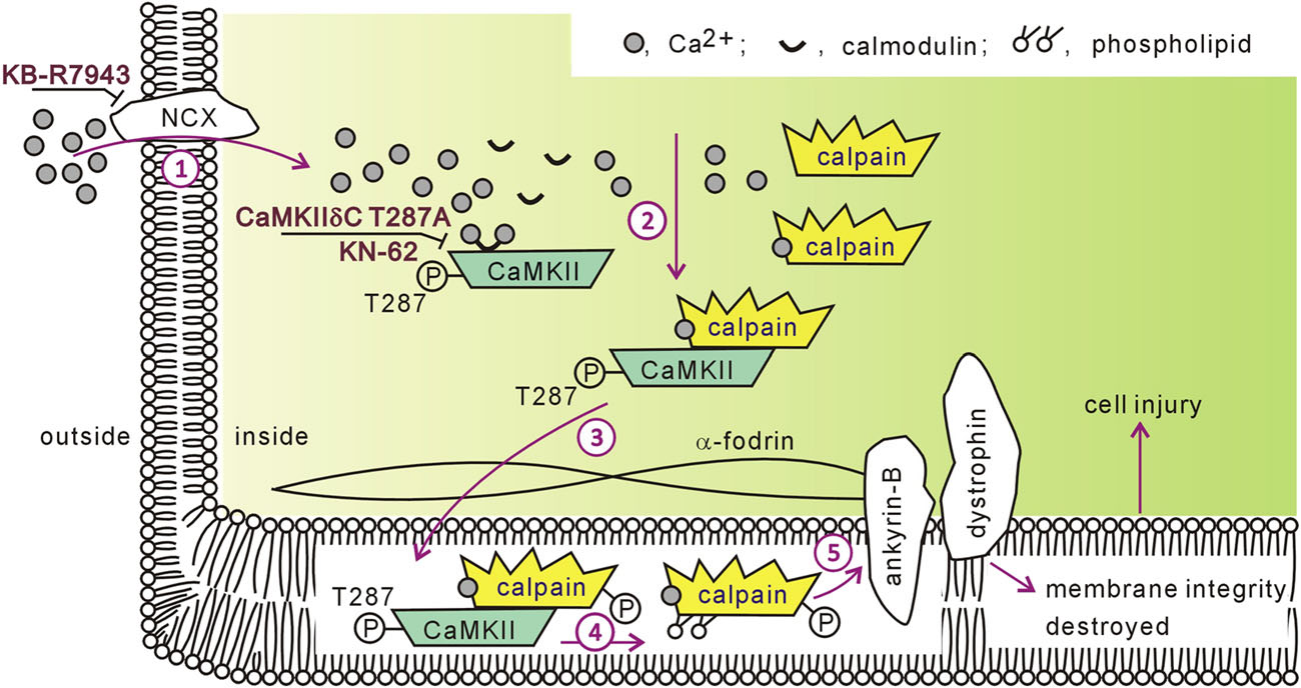
Schematic diagram illustrating the CaMKII/calpain cascade in myocardial ischemia/reperfusion injury. (1) Ca^2+^ influx via the Na^+^/Ca^2+^ exchanger (NCX) triggers intracellular Ca^2+^ overload; (2) Ca^2+^/calmodulin complex stimulates CaMKII auto-phosphorylation at residue T287. CaMKII, which acquires kinase activity, binds to calpain; (3) CaMKII carries its substrate calpain to the sarcolemmal membrane; (4) phospholipid accelerates calpain activation, highly likely with the involvement of phosphorylation; (5) over-activated calpain cleaves membrane skeleton proteins, including α-fodrin, ankyrin-B and dystrophin, and destroys membrane integrity, consequently causing cell injury. KB-R7943 inhibits Na^+^/Ca^2+^ exchanger activity. CaMKIIδC T287A and KN-62 blunt Ca^2+^/calmodulin-elicited CaMKII activation.

The CaMKII/ryanodine receptor cascade is considered the final instigator of intracellular Ca^2+^ overload-induced injury^8,11,12^. CaMKII phosphorylates the ryanodine receptor and provokes aberrant Ca^2+^ to leak from the SR, subsequently leading to hypercontracture, mitochondrial dysfunction, and sarcolemmal rupture^8,11,12^. We previously demonstrated that CaMKII activates calpain, another culprit factor involved in ischemia/reperfusion injury^10^. Thus, it is important to clarify the regulatory effect of CaMKII on calpain, which may provide insight into the mechanism of myocardial ischemia/reperfusion injury. Here, we demonstrated that Na^+^/Ca^2+^ exchanger inhibitor KB-R7943 and CaMKII inhibitor KN-62 protected hearts against Ca^2+^ paradox-induced injury, which was accompanied by decreased calpain activity. Our results also revealed that combined treatment with thapsigargin and ryanodine had no detectable effects. These results support the conclusion that Ca^2+^ influx/CaMKII is an important trigger for calpain activation and that Ca^2+^ leak via the ryanodine receptor is not an obligatory element in CaMKII-induced calpain activation. More importantly, our data demonstrated that: (1) calpain bound to phospho-CaMKII in both Ca^2+^ paradoxical and ischemic/reperfused hearts, the effect of which was negated by the CaMKII inhibitor KN-62, Na^+^/Ca^2+^ exchanger inhibitor KB-R7943, and mutant CaMKIIδC T287A; and (2) CAPN1 membrane recruitment was dependent on phospho-CaMKII in ischemic/reperfused hearts. Collectively, these results provide the first evidence that CaMKII/calpain interaction represents a new pathway mediating intracellular Ca^2+^ overload-induced injury. A schematic figure illustrating the CaMKII/calpain cascade in ischemia/reperfusion injury is shown in Fig. 8.

Calpain is usually present in an inactive state in the cytosol. Our data revealed an increase of CAPN1 in the sarcolemmal membrane, in parallel with a rise of its activity and substrate α-fodrin degradation in ischemic/reperfused hearts. These results are consistent with the observation from our earlier study as well as others, supporting a culprit role for calpain in cardiac injury^10,16,39^. The regulation of calpain activity remains an enigma. Ca^2+^ is not the unique factor governing calpain activity, as Ca^2+^ concentrations required for its proteolytic activity in vitro are much higher than the 50–300 nM Ca^2+^ concentrations that exist during the diastolic period^13^. The results of the present investigation suggest that CAPN1 was recruited to the sarcolemmal membrane, supporting the conclusion that membrane phospholipids lower the Ca^2+^ concentration required for its activation^40,41^. More importantly, both mutant CaMKIIδC T287A and the CaMKII inhibitor KN-62 decreased calpain membrane localization and its activity in ischemic/reperfused hearts. These results provide evidence that CaMKII represents a new molecule that regulates calpain activity.

The Ca^2+^ paradox is a representative scenario that examines Ca^2+^ overload-induced cardiac injury. In the present study, we demonstrated that both Na^+^/Ca^2+^ exchanger inhibitor KB-R7943 and CaMKII inhibitor KN-62 were capable of reversing calpain activation to rescue the heart from the Ca^2+^ paradox. These results revealed that Ca^2+^ paradox shares similarities with ischemia/reperfusion injury, in which Ca^2+^ influx/CaMKII/calpain evoked heart injury^4,5,10^. Conversely, the Ca^2+^ paradox is different from ischemia/reperfusion injury in some respects. Ca^2+^ paradox causes a marked increase in cell necrosis, which manifests as LDH release and TTC staining. Our data showed that both thapsigargin and ryanodine failed to protect hearts against Ca^2+^ paradox-induced injury, which has been reported by a previous study^28^. Therefore, we postulate that the Ca^2+^ leak via the ryanodine receptor is not an obligatory element in CaMKII-elicited calpain activation.

KB-R7943 is a widely used inhibitor of Na^+^/Ca^2+^ exchanger. Here, application of KB-R7943 confirmed the notion that Ca^2+^ influx through Na^+^/Ca^2+^ exchange serves as an important trigger for intracellular Ca^2+^ overload during ischemia/reperfusion^4,5^. More importantly, our data illustrated that Ca^2+^ influx via Na^+^/Ca^2+^ exchanger is an important determinant for CaMKII activation. CaMKIIδ is the predominant cardiac isoform^9^, and at least two splice variants of CaMKIIδ are detected in the heart. One is a δB isoform, which localizes to the nucleus. The other variant is a δC isoform, which has 11 amino acid deletions and exists in the cytosol^9^. In the present investigation, CaMKIIδC T287A reduced CaMKII activity and alleviated heart injury, compared with the AAV vector group and ischemia/reperfusion group. These results have strengthened the concept that CaMKIIδC mediates ischemia/reperfusion injury^30^. A previous study demonstrated that basal activated CaMKII is concentrated at the Z lines^42^. Our data revealed that autophosphorylated CaMKII was recruited to the sarcolemmal membrane in ischemic/reperfused myocardial cells. These results reinforce the conclusion that spatial localization determines the function of CaMKII.

CaMKII is an important kinase and auto-phosphorylation at Thr287 functions as a prerequisite for kinase activation^9^. Here, the mutant CaMKII T287A reduced the interaction between phospho-CaMKII and CAPN1 but did not increase the interaction between CaMKII and CAPN1. The data did not favor a major role for CaMKII T287A mutant in competitively antagonizing the binding between endogenous CaMKII with CAPN1. In this study, we suffered from the technical difficulty of separating Flag-tagged CaMKII mutant (58 kDa) from endogenous CaMKII (55 kDa) using western blot analysis (Fig. 3b). Therefore, it remains unclear whether the inhibition of the mutant on CaMKII auto-phosphorylation is due to competitively binding with the cell injury initiator Ca^2+^/calmodulin or due to reducing endogenous CaMKII expression in ischemia/reperfusion injury. Even so, the data indicated that CaMKII kinase activity is indispensable to the interaction between CaMKII and CAPN1. Previous studies indicated that several amino acid residues of calpain are phosphorylated by protein kinase A, protein kinase C, and mitogen-activated protein kinase^23–25^. Bioinformatics analysis with PhosphositePlus and Group-based Prediction System V5.0 also suggests that multiple amino acid residues in CAPN1, including Ser232 and Ser379 in domain II (catalytic domain) and Ser538 in domain III (regulation domain), are modifiable by CaMKII. Therefore, the physiological significance of these residues in CAPN1 function warrants further investigation. CaMKII possesses several properties, such as mobility^43^, and serves as a carrier or structural component for substrate transmission^26,27^. Our data showed that self-phosphorylated CaMKII translocated to membrane upon ischemia/reperfusion challenge, and that CaMKII T287A mutation did not increase the level of CaMKII in the plasma membrane. These results suggest that CaMKII is mobility dependent upon its kinase activity. In our study, self-phosphorylated CaMKII and CAPN1 were translocated to the membrane upon ischemia/reperfusion insult, and the translocation of CAPN1 to the membrane was diminished with CaMKIIδC T287A. These observations depicted that CaMKII likely functions as a carrier, thus promoting CAPN1 membrane translocation in ischemic/reperfused hearts.

Loss of membrane integrity is a salient feature in injured myocytes^44^, with a role identified for CaMKII in this process. CaMKII activates calpain, which destroys cell structure by cleaving membrane skeleton proteins, such as α-fodrin, ankyrin-B, and dystrophin^10^. In this work, both auto-phosphorylated CaMKII and CAPN1 were recruited to the myocardial plasma membrane. CaMKIIδC T287A blocked calpain activation and the proteolysis of α-fodrin, and therefore improved membrane integrity of myocardial cells. Thus, we conclude that CaMKIIδC/calpain causes myocardial cell injury. Previous studies have demonstrated the importance of CaMKII-dependent phosphorylation of the ryanodine receptor in myocardial ischemia/reperfusion injury by way of Ca^2+^ leakage^5,11^. Here, we demonstrated that CaMKII may bind with calpain. Moreover, as calpain destroys the ryanodine receptor in the SR^20–22^, our data suggest that the CaMKII/calpain may be a novel cascade for initiating ischemia/reperfusion injury. This notion is further strengthened by our data indicating that CaMKII/calpain governed Ca^2+^ paradox-induced injury, independent of the ryanodine receptor. It may be speculated that an interaction may exist between CaMKII/calpain and the CaMKII/ryanodine receptor cascade in ischemic/reperfused hearts, thereby engaging interaction among calpain activation, ryanodine receptor abnormality, and SR Ca^2+^ leak, all of which promote cell injury synergistically.

In summary, our data established that Ca^2+^ leak via the ryanodine receptor is not an obligatory element in CaMKII-induced calpain activation and that CaMKII, likely serving as a kinase as well as a carrier, directly activates calpain to disengage membrane integrity. These findings represent a new avenue in myocardial ischemia/reperfusion injury. Further study is warranted to better clarify the mechanisms behind the regulation of calpain by CaMKII, which may provide a new strategy for heart protection.
